# Ultrasound Stimulation Modulates Microglia M1/M2 Polarization and Affects Hippocampal Proteomic Changes in a Mouse Model of Alzheimer's Disease

**DOI:** 10.1002/iid3.70061

**Published:** 2024-11-26

**Authors:** Xinliang Lu, Wenxian Sun, Li Leng, Yuting Yang, Shuting Gong, Qi Zou, Haijun Niu, Cuibai Wei

**Affiliations:** ^1^ Department of Neurology, Xuan Wu Hospital Capital Medical University Beijing China; ^2^ School of Biological Science and Medical Engineering Beihang University Beijing China

**Keywords:** Alzheimer's disease, cell phenotype, microglia, proteomics, ultrasound stimulation

## Abstract

**Background:**

The effectiveness of ultrasound stimulation in treating Alzheimer's disease (AD) has been reported in previous studies, but the underlying mechanisms remain unclear. This study investigated the effects of ultrasound stimulation on the proportion and function of microglia of different phenotypes, as well as on the levels of inflammatory factors. Additionally, it revealed the alterations in proteomic molecules in the mouse hippocampus following ultrasound stimulation treatment, aiming to uncover potential new molecular mechanisms.

**Methods:**

Ultrasound stimulation was used to stimulate the hippocampus for 30 min per day for 5 days in the ultrasound stimulation‐treated group. Amyloid plaque deposition was measured using immunofluorescence staining. M1 and M2 type microglia were labeled using immunofluorescent double staining, and the ratio was calculated. The levels of Aβ42, IL‐10, and TNF‐α were determined using ELISA kits. The quantitative proteomics method was employed to explore molecular changes in hippocampal proteins.

**Results:**

Ultrasound stimulation treatment reduced the average fluorescence intensity of amyloid plaques and the concentration of Aβ42. Compared to the AD group, ultrasound stimulation resulted in a 14% reduction in the proportion of M1 microglia and a 12% increase in the proportion of M2 microglia. The concentration of the anti‐inflammatory factor IL‐10 was significantly increased in the ultrasound stimulation‐treated group. Proteomics analysis revealed 753 differentially expressed proteins between the ultrasound stimulation‐treated and AD groups, with most being enriched in the oxidative phosphorylation pathway of mitochondria. Additionally, the activity of cytochrome c oxidase, involved in oxidative phosphorylation, was increased after ultrasound stimulation treatment.

**Conclusions:**

Ultrasound stimulation affects microglial polarization, reduces amyloid plaque load, and enhances levels of anti‐inflammatory factors in APP/PS1 mice. Proteomics analysis reveals molecular changes in hippocampal proteins after ultrasound stimulation treatment. The mechanism behind ultrasound stimulation‐induced modulation of microglial polarization may be related to changes in mitochondrial oxidative phosphorylation.

## Graphical abstract

1. Ultrasound reduces the proportion of M1‐type microglia and increases that of M2‐type microglia in APP/PS1 mice 2. Ultrasound induces hippocampal proteomics changes in APP/PS1 mice 3. Ultrasound modulates microglial M1/M2 polarization is related to the pathway of oxidative phosphorylation.

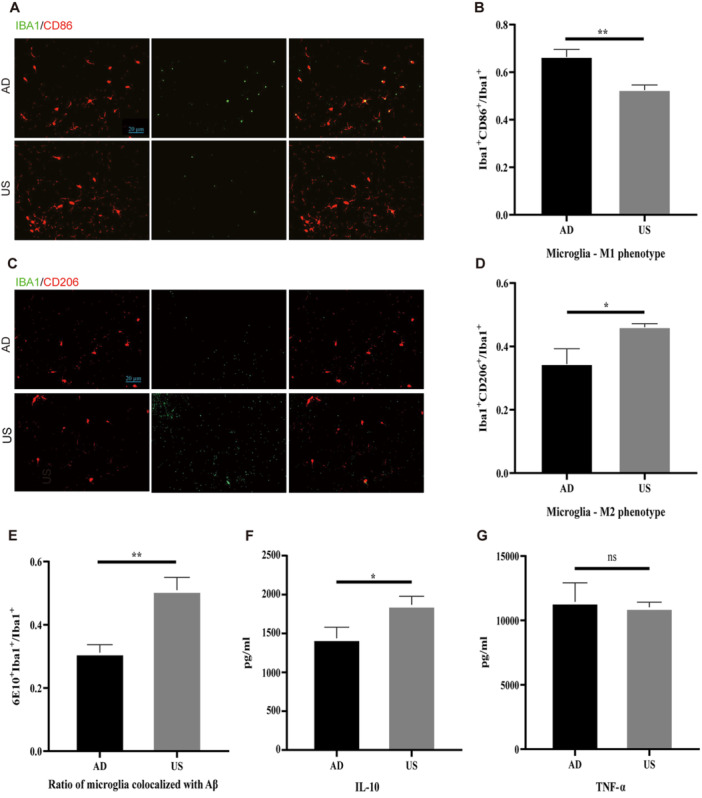

## 1 Introduction

Alzheimer's disease (AD) is a neurodegenerative disorder marked by the accumulation of amyloid beta (Aβ) plaques and neurofibrillary tangles of hyperphosphorylated tau proteins [1]. The prevailing amyloid cascade hypothesis suggests that amyloid accumulation is the primary driver of neuronal damage in AD [2, 3]. However, most clinical trials against AD focused on the amyloid hypothesis have failed [4]. Increasing evidence points to a crucial link between neuroinflammation and AD pathophysiology [5, 6]. Neuroinflammation is increasingly recognized as a significant pathogenic mechanism in AD.

Microglia, the resident immune cells of the central nervous system, play a crucial role in the pathogenesis and progression of AD by mediating neuroinflammation [7, 8]. The polarization of microglia into different phenotypes, each with distinct responses, is a central feature of disease progression [9]. Microglia activation can be classified into a pro‐inflammatory M1 state and an anti‐inflammatory M2 state [10]. As key regulators of inflammation in the central nervous system [11], inhibiting M1‐type polarization or promoting M2‐type polarization of microglia can effectively reduce pro‐inflammatory factors [12] and mitigate pathological damage in AD [13]. Therefore, regulating the M1/M2 polarization of microglia is crucial for the progression and development of AD.

Noninvasive brain stimulation techniques have become pivotal in research for the treatment of neurological disorders. Among these techniques, ultrasound stimulation stands out due to its higher spatial resolution, targeting precision, and penetration depth [14, 15, 16]. Currently, most studies on ultrasound focus on its efficacy in combination with microbubbles to open the blood‐brain barrier for targeted drug delivery [17, 18, 19]. Although some animal studies suggest that this method may alleviate AD pathology, opening the blood‐brain barrier can lead to adverse effects such as inflammation [20]. To ensure the safety of ultrasound as a noninvasive stimulation method, some researchers have shifted focus to exploring the therapeutic effects of ultrasound stimulation without the use of microbubbles. Studies using animal models of AD have shown that ultrasound stimulation can reduce amyloid plaque deposition and improve cognitive dysfunction [21, 22, 23, 24]. Additionally, some clinical trials with small sample sizes have indicated that ultrasound stimulation can enhance cognitive function in individuals with AD [25]. Low‐intensity pulsed ultrasound stimulation has been found to attenuate LPS‐induced neuroinflammation and memory deficits by modulating TLR4/NF‐κB signaling and CREB/BDNF expression [26]. Given the crucial role of microglia in the neuroinflammatory pathology of AD, it remains unclear whether ultrasound stimulation therapy can impact microglial activity in the AD brain. The aim of this study was to investigate whether ultrasound stimulation could regulate microglial polarization and induce proteomic changes in the hippocampus, and to explore potential new mechanisms underlying the beneficial effects of ultrasound stimulation in AD.

## 2 Materials and Methods

### 2.1 Animals

This study was approved by the Animal Ethics Committee of the Beijing Institute of Traditional Chinese Medicine (approval number: 2021030202). The present study followed the ARRIVE guidelines for reporting of animal research (https://www.nc3rs.org.uk/arrive-guidelines). Male APP/PS1 and C57BL/6 mice aged 6 months (25–29 g) were obtained from Beijing Huafukang Biotechnology (license number: SCXK 2019‐0008; Beijing, China). All mice were housed in a controlled environment with a temperature of 20 ± 2℃, humidity maintained at 60%, and subjected to a 12‐h light/12‐h dark cycle. Mice were grouped according to random digits table generated by computer software. The mice were divided into three groups: a normal control sham treatment group (NC group; C57BL/6, *n* = 11), an AD model sham treatment group (AD group; APP/PS1, *n* = 11), and an AD model ultrasound stimulation treatment group (US group; APP/PS1, *n* = 11). Only mice that survived the entire experimental procedure were included in the analysis and those that died in the middle of experiments due to surgical complications were not counted.

### 2.2 Ultrasound Stimulation Equipment and Treatment

**Figure 1:**
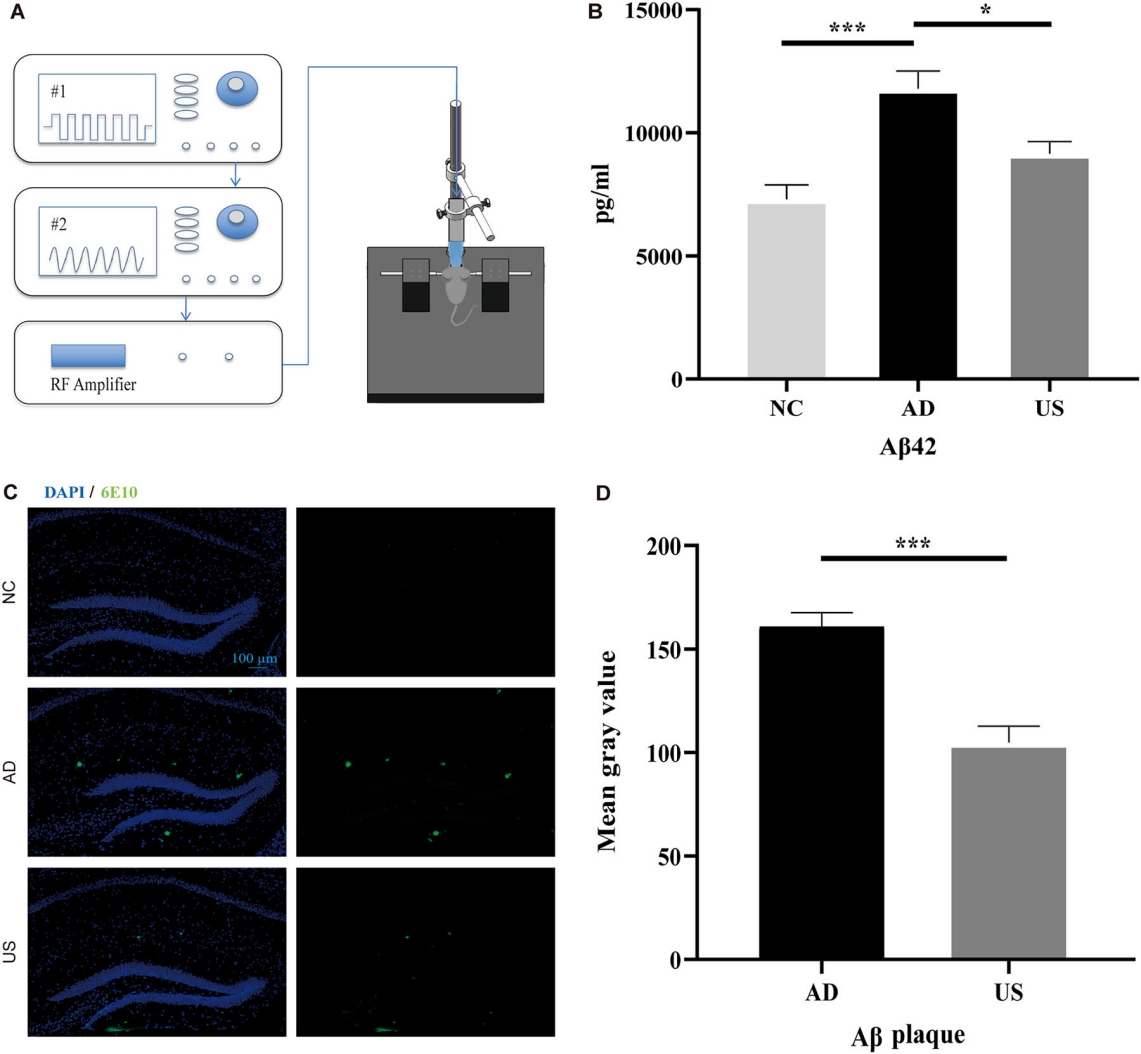
Ultrasound stimulation reduced Aβ pathology in APP/PS1 mice. (A) Schematic diagram of the ultrasound stimulation treatment platform. (B) Aβ42 content in the hippocampus as determined by ELISA (*n* = 5 per group). (C) Representative images of immunostaining for amyloid plaques in the hippocampus of the normal control (NC) and sham treated (AD), and ultrasound stimulation treated (US) Alzheimer's disease groups. Scale bar, 100 mm. (D) Mean gray value of amyloid plaques in the AD and US groups (*n* = 6 per group). *p < 0.05, **p < 0.01, ***p < 0.001.

A schematic diagram of the ultrasound stimulation treatment platform is shown in Figure 1A. The device amplifies the waveform from a transducer (V303‐SU; Olympus) by a power amplifier (1040 L; E&I) to emit ultrasonic waves. The transducer is excited by two signal generators (AFG3252, AFG3021B; Tektronix). In ultrasound stimulation parameters, frequency, sound intensity, and pulse repetition frequency play a key role. The attenuation coefficient of biological tissue is nearly proportional to the ultrasound frequency, so this study employed a frequency of 1 MHz, a parameter that has also been widely used in most brain ultrasound stimulation studies [21, 27]. To ensure the safety of the animals and stay within the energy output range of the ultrasound equipment, the excitation voltage was set at 330 mVpp, and Ispta was calculated to be 294 mW/cm² based on measured sound pressure. Since 40 Hz gamma oscillations have been shown to play a positive role in clearing Aβ plaques [28], the PRF was set to 40 Hz. The remaining parameters were set as follows: DC = 10%, SD = 5 s, ISI = 5 s. Mice were anesthetized with isoflurane (3% for induction and 1.5% for maintenance) using a gas anesthesia machine (R630; RWD) and fixed in a mouse stereotaxic apparatus. A heating pad was used to maintain body temperature. Mice in the US group were shaved, and the ultrasonic probe coated with ultrasonic coupling agent was targeted at the hippocampus (1.8 mm posterior, 1 mm lateral to Bregma) for 30 min per day (15 min on the left and 15 min on the right side) for 5 days. Mice in the sham treatment group were shaved and anesthetized in the same way as mice in the US group but ultrasound stimulation placed on the head was not turned on. After treatment, the mice were returned to their cages and allowed to recover from anesthesia.

### 2.3 Immunofluorescence Staining and ELISA

After treatment, 6 mice in each group were anesthetized with pentobarbital sodium (1%, 50 mg/kg) via intraperitoneal injection. Brain tissues were collected after cardiac perfusion. The brain tissues were soaked in 4% paraformaldehyde at 4℃ for 48 h, embedded in paraffin, and cut into 5‐μm sections. The sections were incubated with a mouse anti‐6E10 antibody (1:500, 803004; Covance) at 4℃ overnight and then with a fluorescein isothiocyanate‐conjugated goat anti‐mouse secondary antibody (1:50, SA00003‐1; Proteintech) for Aβ detection at room temperature for 1 h.

Immunofluorescence double staining was used to observe different microglial phenotypes. Tissue sections were incubated with a rabbit anti‐Iba1 antibody (1:500, 019‐19741; Wako) and a rabbit anti‐CD86 (M1 market; 1:500, ab119857; Abcam) or anti‐CD206 antibody (M2 market; 1:5000, 60143‐1‐IG; Proteintech). After the second antibody incubation, the sections were coverslipped with anti‐fluorescence attenuator containing 4′,6‐diamidino‐2‐phenylindole (ab104139, Abcam) and observed under a fluorescence microscope (Axio Imager.M2; Zeiss). The average fluorescence intensity and fluorescence colocalization coefficient were calculated based on images, using the ImageJ software.

After treatment, five mice in each group were killed by cervical dislocation, and the brains were quickly collected on ice and placed in liquid nitrogen for cryopreservation. The contents of Aβ1‐42, IL‐10, and TNF‐α, were determined using ELISA kits (Aβ1‐42, CSB‐E10787m; IL‐10, CSB‐E04594m; TNF‐α, CSB‐E04741m; Cusabio) per the manufacturer's instructions.

### 2.4 Determination of Mitochondrial Respiratory Chain Complex IV (Cx IV) Activity

Cx IV, also known as cytochrome c oxidase, is responsible for the oxidation of reduced cytochrome c that has characteristic light absorption features at a wavelength of 550 nm. Cx IV activity was determined using a Microglial Mitochondrial Respiratory Chain Complex IV Activity Assay Kit (BC0945; Solarbio).

### 2.5 Quantitative Proteomics Based on TMT

#### 2.5.1 Protein Sample Preparation

Samples (*n* = 3 per group) and working fluid (RIPA lysis buffer with protease inhibitors) were thoroughly mixed and fully dissolved by sonication followed by centrifugation at 14000 × *g*, 4℃ for 15 min. The supernatants were transferred to fresh Eppendorf tubes. Protein concentrations were determined using a bicinchoninic acid assay kit (Thermo Scientific) according to the manufacturer's instruction. One hundred milligram of protein was mixed with the disulfide bond‐reducing agent tris (2‐carboxyethyl) phosphine for protein reduction and then, indole acetic acid reagent was added to alkylate the reduced disulfide bonds. The proteins were then precipitated using acetone, redissolved, and digested with trypsin to form peptides for TMT labeling per the manufacturer's instruction. Then, trifluoroacetic acid was added to precipitate the sodium deoxycholate followed by centrifugation. The supernatants were transferred to fresh Eppendorf tubes to obtain the labeled polypeptide sample. Then, the samples were desalted using a C18 desalting column (SPE cartridges), vacuum‐dried, and the polypeptides were redissolved in aqueous acetonitrile formic acid for reversed‐phase‐ reversed‐phase fractionation. One hundred micrograms of TMT‐labeled polypeptides were separated by reversed‐phase high performance liquid chromatography at pH = 10. One fraction was collected every 30 s, and a total of 120 fractions were collected and combined at 10‐s intervals to obtain 12 fractions. The samples were vacuum‐dried and stored at –80℃ before liquid chromatography tandem mass spectrometry (LC‐MS/MS) analysis.

#### 2.5.2 Liquid Chromatography Tandem Mass Spectrometry

For each fraction, 2 μg of peptides was separated and analyzed using nano‐UPLC (EASY‐nLC1200) and Q‐Exactive mass spectrometry. Samples were loaded onto a 100‐μm ID × 15 cm reverse column (ReproSil‐Pur 120 C18‐AQ, 1.9 μm; Dr. Maish) using an autosampler. The chromatographic column was equilibrated with 100% buffer A, and then buffer B was used for gradient separation at a flow rate of 300 nL/min for 90 min. Buffer A was a 0.1% formic acid, 2% acetonitrile aqueous solution and buffer B a 0.1% formic acid, 80% acetonitrile aqueous solution. The samples were separated using an easy‐NLC1200 chromatograph and analyzed using Q‐Exactive mass spectrometer. Samples were analyzed in the positive ion mode for 120 min, and the scanning range of precursor ion was 350–1600 m/z. Twenty fragments were collected after each full scan (MS2 Scan).

#### 2.5.3 Database Search

LC‐MS/MS raw data were searched and quantitatively analyzed using the protein sequence database using MaxQuant (v. 1.5.6.0). Fold change values and P‐values of different protein contents between groups were obtained. The parameters used in the MaxQuant database search are shown in Table S1 (Supplementary Materials). Proteins meeting the conditions of fold change > 1.2 or < 1/1.2, *p* < 0.05, and unique peptide > 2 were extracted as differentially expressed proteins (DEPs) for bioinformatics analysis.

#### 2.5.4 Bioinformatics Analysis

A hierarchical clustering map and volcano plot were generated to visualize the DEPs. Gene Ontology (GO) analysis was performed to examine the enrichment of DEPs in biological process (BP), Cellular Component (CC), and molecular function (MF) categories. Moreover, classical signaling pathways associated with DEPs were identified through Kyoto Encyclopedia of Genes and Genomes (KEGG) analysis. The clustering was executed using the clusterProfiler package (R v. 3.6.1), while GO analysis and KEGG analyses were conducted using the DAVID platform (https://david.ncifcrf.gov/).

### 2.6 Statistics

Statistical analysis was performed using GraphPad Prism 8.0 software. The data are presented as mean ± SEM and were analyzed using Student's t‐test or one‐way ANOVA. Significance was accepted at *p* < 0.05.

## 3 Results

### 3.1 Ultrasound Stimulation Alleviates Aβ Pathology in APP/PS1 Mice

The concentration of aggregation‐prone Aβ42 was measured in 6‐month‐old mice, which were either treated with ultrasound stimulation (US group) for 30 min daily over 5 days or left untreated (sham treatment group, AD and NC group). The Aβ42 concentration in the hippocampus was lowest in the NC group (6435 ± 1011.90 pg/mL) and highest in the AD group (12086 ± 1466.63 pg/mL). The US group showed a significantly lower concentration of Aβ42 (9251 ± 737.30 pg/mL) compared to the AD group (*p* < 0.05, Figure 1B). Immunofluorescence staining results revealed evident amyloid plaques in both the AD and US groups' hippocampi (Figure 1C), but the fluorescence intensity was significantly lower in the US group (104 ± 21.67) than in the AD group (154 ± 12.93; *p* < 0.001; Figure 1D), indicating fewer amyloid plaques in the US group.

### 3.2 Ultrasound Stimulation Modulates Microglial Polarization of M1/M2 in APP/PS1 Mice

**Figure 2:**
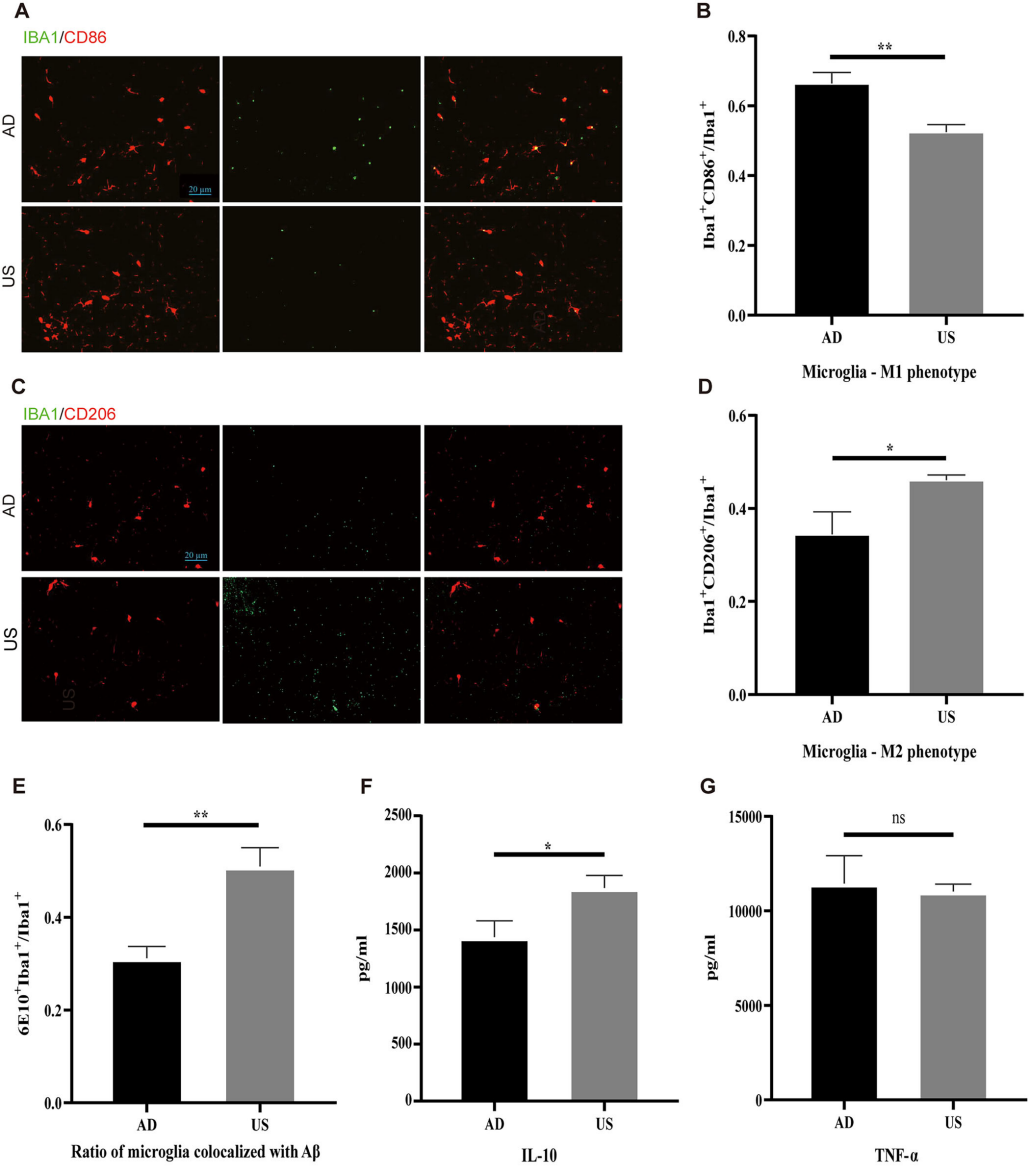
Ultrasound stimulation promotes microglial polarization from M1 to M2. (A) Representative images of co‐immunostaining for Iba1 and CD86 in the hippocampus of sham treated (AD) and ultrasound stimulation treated (US) Alzheimer's disease groups (*n* = 6 per group). Scale bar, 20 μm. (B) The colocalization coefficient of microglia and an M1 marker (CD86) was measured. (C) Representative images of co‐immunostaining for Iba1 and CD206 in the hippocampus of sham treated (AD) and ultrasound stimulation treated (US) Alzheimer's disease groups (*n* = 6 per group). (D) The colocalization coefficient of microglia and an M2 marker (CD206) was measured. (E) The colocalization coefficient of microglia and amyloid plaques. (F) IL‐10 level and (G) TNF‐α level in the hippocampus as determined by ELISA (*n* = 5 per group). *p < 0.05, **p < 0.01.

In the AD group, there were significantly more M1 microglia in the hippocampus compared to the US group. The colocalization coefficient of microglia with the M1 marker CD86 was significantly higher in the AD group than in the US group (66.4% ± 7.78% vs. 52.5% ± 5.19%, *p* < 0.01; Figure 2A,B). Conversely, the colocalization coefficient of microglia with the M2 marker CD206 was significantly lower in the AD group than in the US group (34.4% ± 11.85% vs. 46.1% ± 2.68%, *p* < 0.05; Figure 2C,D).

### 3.3 Ultrasound Stimulation Enhances Microglial amyloid‐β Clearance in APP/PS1 Mice

The polarization of microglia from M1 to M2 is associated with enhanced phagocytic ability, indicated by the colocalization ratio of microglia and amyloid plaques. The proportion of microglia colocalizing with amyloid plaques was significantly higher in the US group compared to the AD group (50.55% ± 3.58% vs. 31.17% ± 2.55%, *p* < 0.01; Figure 2E).

### 3.4 Ultrasound Stimulation Increases the Levels of Anti‐Inflammatory Factors in APP/PS1 Mice

Microglial polarization from M1 to M2 affects downstream cytokine levels, leading to a decrease in inflammatory factors and an increase in anti‐inflammatory factors. We found that the concentration of the anti‐inflammatory factor interleukin‐10 (IL‐10) was significantly higher in the US group compared to the AD group (1874.68 ± 131.14 pg/mL vs. 1335.83 ± 185.61 pg/mL, *p* < 0.05; Figure 2F). There was no significant difference in the concentration of the inflammatory factor TNF‐α between the groups (Figure 2G).

### 3.5 Proteomics Changes in the Hippocampus of APP/PS1 Mice After Ultrasound Stimulation Treatment

#### 3.5.1 Differentially Expressed Hippocampal Proteins Between AD and CN Mice

**Figure 3:**
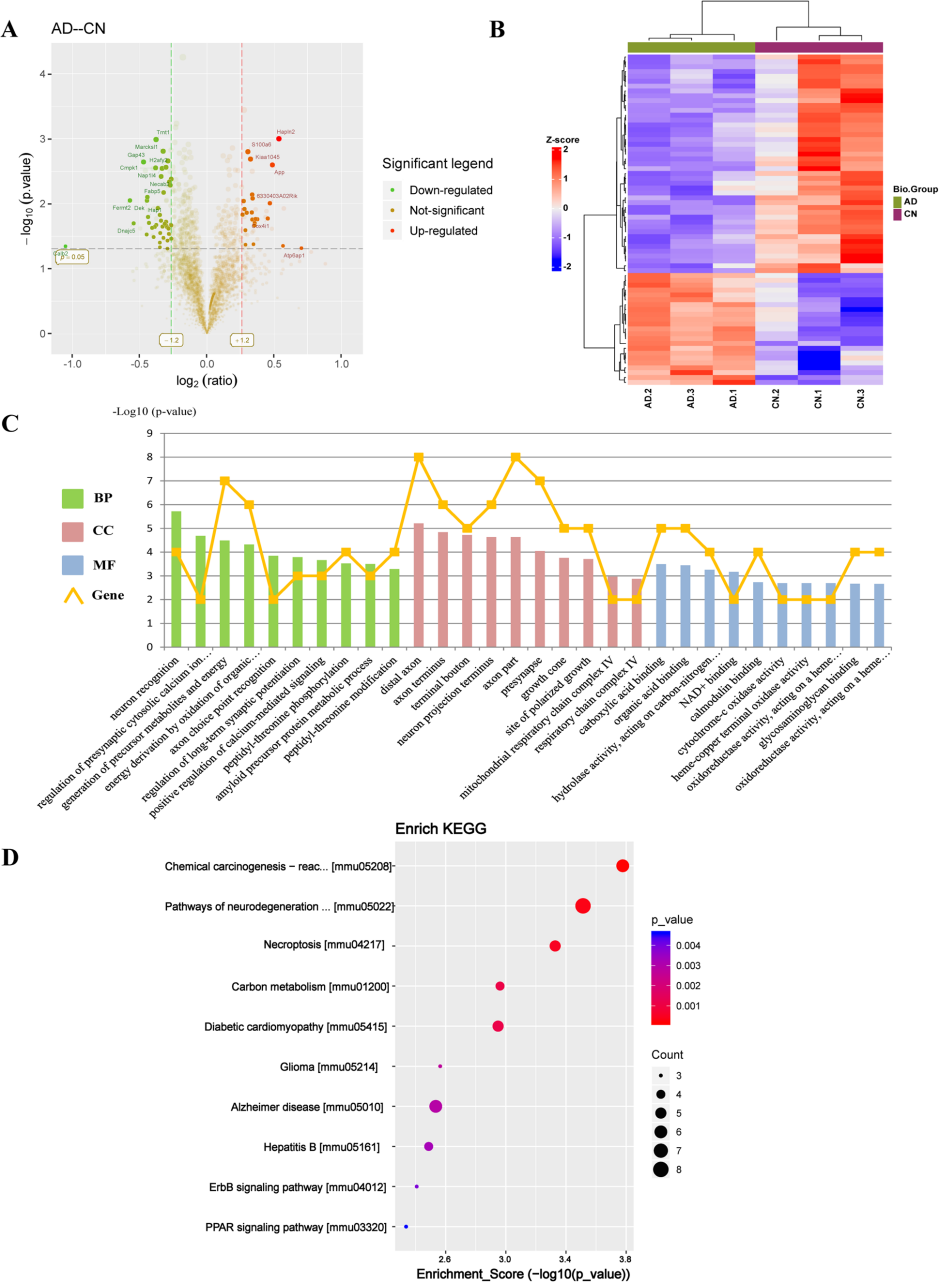
Bioinformatics analysis of DEPs between the AD and NC groups. (A) Volcano plot of DEPs between the AD and NC groups. Downregulated and upregulated proteins are shown in green and red, respectively. (B) Heatmap of the DEPs. (C) GO enrichment analysis of DEPs between the AD and NC groups. BP (green), CC (red), and MF (blue) are ranked according to p‐value, and the ten most significantly enriched terms are displayed. The orange graph indicates the number of genes in each term. (D) KEGG enrichment analysis of DEPs between the AD and NC groups. The ten most significantly enriched classical signaling pathways are shown according to *p* value. Dot color represents the *p* value and dot size represents the number of genes in each pathway.

In the study, 17,077 peptides and 3650 proteins were identified from the UniProt mouse database, of which 3593 proteins could be quantified. Between the AD and CN groups, 68 DEPs were identified: 23 upregulated and 45 downregulated. A volcano plot illustrates the upregulated and downregulated DEPs between the AD and CN groups (Figure 3A). Cluster analysis confirmed the reliability of the subsequent analyses by showing tight clustering within groups and clear separation between groups (Figure 3B).

GO enrichment analysis (Figure 3C) indicated that the BP associated with DEPs included neuron recognition, regulation of presynaptic cytosolic calcium ion concentration, generation of precursor metabolites and energy, and energy derivation by oxidation of organic compounds, etc. The DEPs were enriched in CC such as the distal axon, axon terminus, terminal bouton, neuron projection terminus and mitochondrial respiratory chain complex IV, etc. In terms of MF, DEPs were enriched in carboxylic acid binding, organic acid binding, NAD+ binding, and cytochrome‐c oxidase activity, etc. KEGG pathway enrichment analysis (Figure 3D) revealed that the 68 DEPs were predominantly associated with chemical carcinogenesis‐reactive oxygen species, neurodegenerative pathway‐multiple diseases, necroptosis, and Alzheimer disease, etc.

#### 3.5.2 Differentially Expressed Hippocampal Proteins Between the US‐Treated and AD Mice

**Figure 4:**
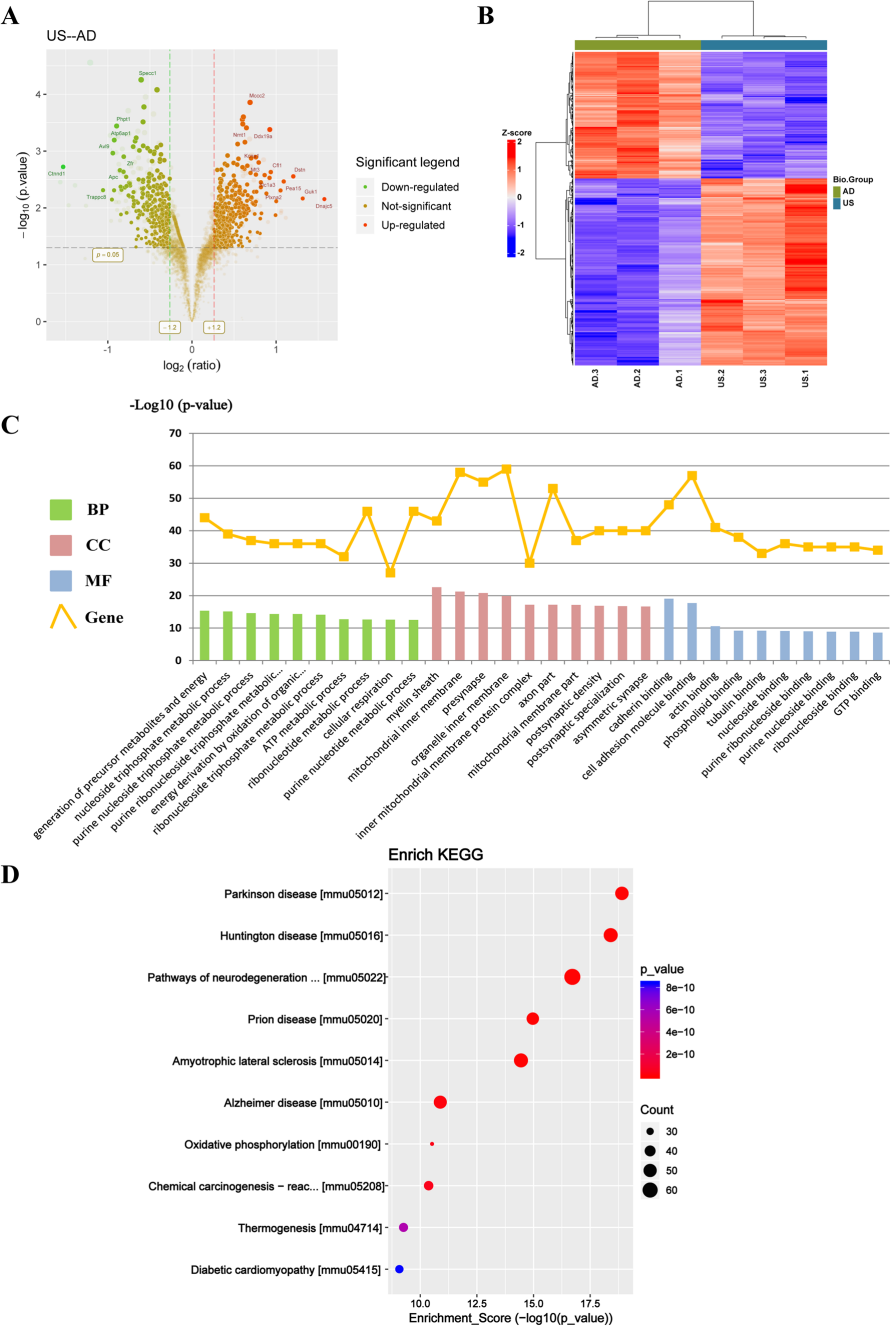
Bioinformatics analysis of DEPs between the US and AD groups. (A) Volcano plot of DEPs between the US and AD groups. Downregulated and upregulated proteins are shown in green and red, respectively. (B) Heatmap of the DEPs. (C) GO enrichment analysis of DEPs between the US and AD groups. BP (green), CC (red), and MF (blue) are ranked according to *p* value, and the ten most significantly enriched terms are displayed. The orange graph indicates the number of genes in each term. (D) KEGG enrichment analysis of DEPs between the US and AD groups. The ten most significantly enriched classical signaling pathways are shown according to *p* value. Dot color represents the *p* value and dot size represents the number of genes in each pathway.

Relative changes in protein levels between the US‐treated and AD groups are shown in a volcano plot (Figure 4A). There were 753 DEPs identified in the US group compared to the AD group, with 449 upregulated and 304 downregulated. A heatmap of the DEPs is presented in Figure 4B.

The 753 DEPs were analyzed for GO term enrichment, focusing on BP, CC, and MF. The 10 most significantly enriched terms in each category were shown in Figure 4C. In the BP category, DEPs were mainly involved in the generation of precursor metabolites and energy, nucleoside triphosphate, purine nucleoside and ATP metabolic processes. For CC, DEPs were associated with the myelin sheath, mitochondrial inner membrane, inner mitochondrial membrane protein complex, and postsynaptic density. In the MF category, DEPs were primarily involved in cadherin, actin, tubulin and purine nucleoside binding. KEGG pathway enrichment analysis highlighted the 10 most significantly enriched pathways in a bubble diagram (Figure 4D). The DEPs were mainly related to Parkinson's disease, Huntington's disease, pathways of neurodegeneration–multiple diseases, Alzheimer's disease, and oxidative phosphorylation.

### 3.6 Ultrasound Stimulation Affects the Function of the Mitochondrial Respiratory Chain in the “Alzheimer's Disease” Pathway

**Figure 5:**
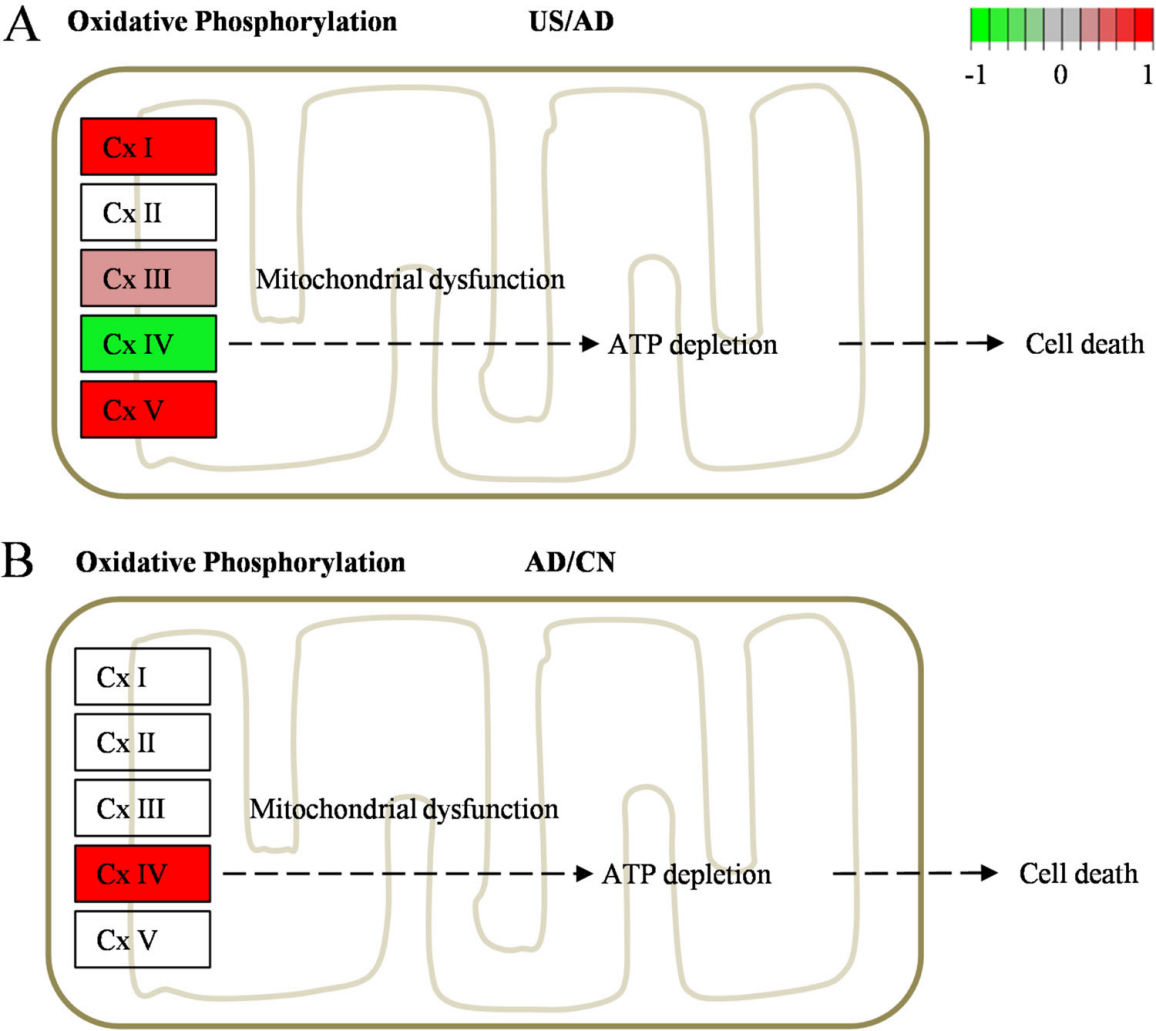
Alterations in mitochondrial respiratory chain CxIV in the “Alzheimer's disease” pathway. (A) Cx IV expression was downregulated in the US group compared to the AD group. (B) Cx IV expression was upregulated in the AD group compared to the NC group.

The NADH oxidative respiratory chain, responsible for ATP production, consists of mitochondrial respiratory chain complexes: Cx I (NADH dehydrogenase), Cx III (coenzyme Q‐cytochrome c reductase), Cx IV (cytochrome c oxidase), and Cx V (ATP synthase). In the “Alzheimer's disease” pathway (Supplementary material 1), DEPs in the US group showed upregulation of Cx I, Cx III, and Cx V, and downregulation of Cx IV versus the AD group (Figure 5A). Cx IV expression was found to be upregulated in the AD group compared to the NC group (Figure 5B). This suggests that ultrasound stimulation therapy may affect Cx IV enzymes in mitochondrial oxidative respiratory chain. Further verification via ELISA revealed that Cx IV activity was highest in the NC group and lowest in the AD group. Ultrasound stimulation treatment significantly increased Cx IV activity (36.64 ± 9.92 U/g in AD vs. 70.72 ± 24.32 U/g in US, *p* < 0.05).

## 4 Discussion

In this study, we investigated the effects of ultrasound stimulation on pathology, neuroinflammation, and hippocampal proteomic changes in APP/PS1 AD mouse models. The findings revealed that ultrasound stimulation influenced microglial polarization and alleviated amyloid pathology in APP/PS1 mice. Further proteomic analysis identified that various proteins were involved in several functions following ultrasound stimulation treatment, including the generation of precursor metabolites and energy, nucleoside triphosphate, purine nucleoside, and ATP metabolic processes. Additionally, we found that ultrasound stimulation‐induced regulation of microglial polarization might be related to the mitochondrial oxidative phosphorylation pathway. Ultrasound stimulation therapy was shown to upregulate the activity of CxIV in the oxidative respiratory chain.

The pathogenesis of AD is related to an imbalance between the inflammatory response mediated by M1 microglia and the anti‐inflammatory activity mediated by M2 microglia [29]. M1 to M2 polarization generally occurs with a decrease in inflammatory factors and an increase in anti‐inflammatory factors. Classical M1‐type microglia tend to express and release inflammatory cytokines, such as IL‐1, iNOS, and TNF‐α, which exacerbate tissue damage. In contrast, M2‐type microglia exhibit neuroprotective effects by secreting anti‐inflammatory cytokines such as IL‐4, IL‐10, and TGF‐β, which attenuate inflammatory responses and promote tissue repair [9, 30]. Therefore, modulation of microglial polarization from M1‐type to M2‐type is crucial for improving AD pathology and cognitive decline. This study is the first to show that ultrasound stimulation modulates the ratio of M1 and M2 microglia in the APP/PS1 AD mouse model, resulting in a decrease in M1 microglia and an increase in M2 microglia.

Studies have shown that ultrasound stimulation treatment reduces Aβ and tau lesions in animal models of AD and promotes hippocampal neurogenesis. It increases nerve growth factor content, activates TRKA‐related survival signaling pathways, and improves cognitive functioning [31, 32]. The benefits of ultrasound stimulation for AD are associated with microglia, reducing IBA‐1‐positive microglia and amyloid plaques [22]. Transcranial ultrasound stimulation enhances autophagy to promote the phagocytosis and degradation of β‐amyloid through the activation of microglial Piezo1, alleviating neuroinflammation, synaptic plasticity impairment, and neural oscillation abnormalities in 5xFAD mice [33]. Research conducted by Bobola et al. demonstrated that ultrasound stimulation increased the co‐localization of activated microglia with amyloid plaques and decreased Aβ load, suggesting that ultrasound stimulation enhances microglial clearance of Aβ [23]. However, it is not clear whether ultrasound stimulation treatment can modulate microglial polarization in the AD brain. A recent cellular experimental study showed that ultrasound stimulation improves microglial polarization in vitro [34], and we confirmed this finding at the animal level.

Previous studies revealed that in lipopolysaccharide‐induced AD mice treated with ultrasound stimulation, the Toll‐like receptor 4/Nuclear factor kappa‐B inflammatory signaling pathway was inhibited in the microglia, leading to decreased expression of the inflammatory cytokine TNF‐α [26]. Consistent with these findings, our study showed that the level of TNF‐α decreased, while the level of the anti‐inflammatory factor IL‐10 significantly increased after ultrasound stimulation treatment. This suggests that ultrasound stimulation enhances the anti‐inflammatory effects of microglia. In our study, ultrasound stimulation promoted the recruitment of microglia to amyloid plaques and increased their colocalization ratio. This finding is supported by another study, which reported a significant increase in the number of microglia colocalizing with amyloid plaques in 6‐month‐old 5XFAD mice treated with ultrasound stimulation for 1 h [23]. Overall, the results of our study suggest that ultrasound stimulation promotes the polarization of microglia from the M1 type to the M2 type in the brains of AD mice, thereby decreasing the levels of pro‐inflammatory factors and increasing the levels of anti‐inflammatory factors.

In this study, proteomic sequencing of the hippocampus in mice showed that the differential proteins between the US and AD groups were significantly enriched in the oxidative phosphorylation pathway. Glycolysis and oxidative phosphorylation, coupled with the tricarboxylic acid cycle, are the main pathways through which ATP is produced to provide energy for cells. These different modalities of energy metabolism are closely related to the microglial phenotype [35, 36, 37]. M2 microglia generally meet their energy requirements through oxidative phosphorylation, whereas M1 microglia mainly rely on glycolysis [38]. Our findings revealed an increase in the proportion of M2‐type microglia in the brains of AD mice after ultrasound stimulation treatment, which may be linked to the oxidative phosphorylation pathway.

Additionally, we observed changes in the mitochondrial respiratory chains within the “Alzheimer's disease” pathways. Ultrasound stimulation primarily induced oxidative phosphorylation in mitochondrial aerobic metabolism, with an upregulation of Cx I, Cx III, and Cx V, and a downregulation of Cx IV. Cx IV, also known as cytochrome c oxidase, transfers electrons from cytochrome C to molecular oxygen and is a key mitochondrial enzyme involved in the oxidative phosphorylation process [39, 40]. Cx IV activity is reportedly low in the brains of AD patients and AD animals, leading to inhibited oxidative mitochondrial energy metabolism and resulting in ATP synthesis disorders [41, 42]. In our study, Cx IV activity in the brain was significantly reduced in the AD group compared to the NC group, and ultrasound stimulation significantly enhanced Cx IV activity in the US group. These results suggest that ultrasound stimulation may enhance oxidative phosphorylation and provide direction for further exploration of the mechanism by which ultrasound stimulation regulates microglial polarization.

This study had some limitations. First, we evaluated the effects of ultrasound stimulation on the hippocampus of APP/PS1 mice based on histopathology and proteomic analysis; however, we did not conduct behavioral experiments. Future research should include behavioral experiments to determine if changes in Aβ equate to changes in cognitive function. Second, we evaluated the expression changes of a limited number of proteins. Further analysis of the expression of other respiratory chain complexes and ATP levels will be necessary to support our hypothesis regarding the mechanism of action of ultrasound stimulation. Thirdly, this study focused on a specific set of ultrasound parameters and did not examine the effects of varying these settings. In future research, we plan to explore a wider range of ultrasound parameters to optimize stimulation protocols and deepen our understanding of their effects. Lastly, the study should be replicated in larger animal models to confirm the broader applicability of our results. This step is crucial for translating our findings into potential clinical applications and ensuring their relevance to human physiology.

## 5 Conclusions

Ultrasound stimulation affects microglial polarization, reduces amyloid plaque load, and enhances levels of anti‐inflammatory factors in APP/PS1 mice. Proteomics analysis reveals molecular changes in hippocampal proteins after ultrasound stimulation treatment. The mechanism behind ultrasound stimulation‐induced modulation of microglial polarization may be related to changes in mitochondrial oxidative phosphorylation.

## Author Contributions

**Cuibai Wei:** conceptualization, supervision, funding acquisition. **Haijun Niu:** supervision, writing–review and editing. **Xinliang Lu:** formal analysis, methodology, writing–original draft. **Wenxian Sun:** data curation, writing–review and editing. **Li Leng:** investigation, methodology. **Yuting Yang:** software. **Shuting Gong:** visualization, validation. All authors have read and agreed to the published version of the manuscript.

## Ethics Statement

This study was approved by the Animal Ethics Committee of the Beijing Institute of Traditional Chinese Medicine (approval number: 2021030202).

## Conflicts of Interest

The authors declare no conflict of interest.

## Supporting information

Supporting information.

Supporting information.

## Data Availability

The data supporting the findings of the article are available on request by contacting the corresponding author.
