# Supplementary figures and images for: Ultrasound Stimulation Modulates Microglia M1/M2 Polarization and Affects Hippocampal Proteomic Changes in a Mouse Model of Alzheimer's Disease
Source: Immun Inflamm Dis. 2024 Nov 26;12(11):e70061. doi: 10.1002/iid3.70061 (PMC11590030; doi:10.1002/iid3.70061)

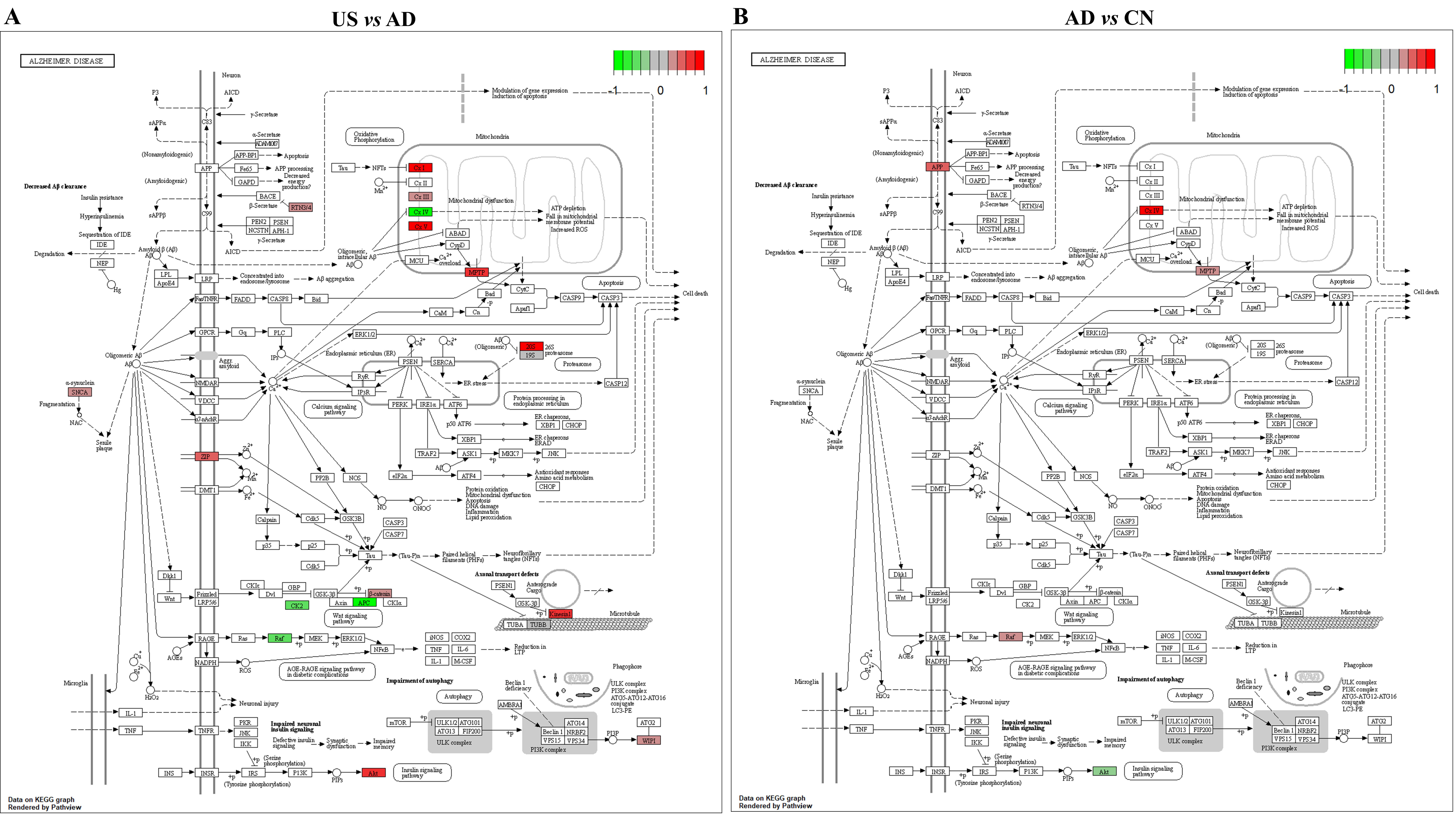

Supplement: Supplementary file 1 — Supporting information. [file IID3-12-e70061-s002.tif]
