# Supplementary material for: Ultrasound Stimulation Modulates Microglia M1/M2 Polarization and Affects Hippocampal Proteomic Changes in a Mouse Model of Alzheimer's Disease
Source: Immun Inflamm Dis. 2024 Nov 26;12(11):e70061. doi: 10.1002/iid3.70061 (PMC11590030; doi:10.1002/iid3.70061)
Supplement: Supplementary file 2 — Supporting information. [file IID3-12-e70061-s001.docx]

**Table S1. Parameters used in the search of MaxQuant database**

| Protein database | uniprot-proteome-mouse-2021.2.fasta |
| --- | --- |
| Specific enzyme | trypsin |
| Maximum number of missed cuts | 2 |
| Quantitative method | TMT 16 plex |
| Variable modification | Oxidation (M) ethodmissed cuts.fastam |
| Fixed modification | Carbamidomethyl (C) |
| Minimum polypeptide length | 7 |
| Maximum peptide molecular weight | 4600 Da |
| Peptide FDR | ≤ 0.01 |
